# Antioxidant, Anti-Inflammatory, and Postulated Cytotoxic Activity of Phenolic and Anthocyanin-Rich Fractions from Polana Raspberry (*Rubus idaeus* L.) Fruit and Juice—In Vitro Study

**DOI:** 10.3390/molecules23071812

**Published:** 2018-07-21

**Authors:** Urszula Szymanowska, Barbara Baraniak, Anna Bogucka-Kocka

**Affiliations:** 1 Department of Biochemistry and Food Chemistry, University of Life Sciences, Skromna Str. 8, 20-704 Lublin, Poland; barbara.baraniak@up.lublin.pl; 2 Chair and Department of Biology with Genetics, Medical University of Lublin, Chodźki Str. 4a, 20-093 Lublin, Poland; anna.kocka@umlub.pl

**Keywords:** raspberry mash and juice, phenolic and anthocyanin-rich fraction, antioxidant activity, anti-inflammatory activity, cytotoxic activity

## Abstract

In this study, the antioxidative and anti-inflammatory potential of crude extracts (CE), anthocyanin-rich fractions (ARF), and phenolic fractions (PF) from raspberry (R) and raspberry juice (J) were evaluated. The antioxidant properties were evaluated with three complementary assays: DPPH radical scavenging activity, chelating Fe(II) power, and ferric reducing power. The highest antioxidant activity was determined for the crude extract from raspberry pulp (RCE) in the case of all methods used. The anti-inflammatory activity was demonstrated by inhibitory effect on lipoxygenase (LOX) and cyclooxygenase-2 (COX-2) activity in vitro. The highest efficiency in inhibiting the activity of both enzymes was exhibited by RCE, 0.79 and 0.59 mg FW/mL, respectively. In turn, JARF had the lowest ability to inhibit LOX (EC50 = 4.5 mg FW/mL) and JPF caused the lowest COX-2 inhibition (1.75 mg FW/mL). Additionally, we have performed a pilot study of in vitro cytotoxic activity using two human leukemia cell lines: J45 and HL60. All examined extracts inhibited the viability of J45 cells more effectively than HL60. The highest cytotoxic effect was observed in the J45.01 cell line after exposure to RCE (EC50 = 0.0375 mg FW/mL).

## 1. Introduction

Berries contain a significant amount of diverse bioactive compounds, which individually or in combination can have a positive effect on human health [1]. Therefore, berry fruit can be recommended as a natural source of antioxidants. Raspberries (*Rubus idaeus* L.) are very popular and attractive fruit from the family *Rosaceae* [1,2]. Poland is one of the world’s top producers of raspberries. In the years 2014–2016, it stood third in the global ranking, only behind Russia and the United States, and ahead of Mexico and Serbia. As with other berries in Poland, most raspberries are processed into jams, juices, jellies, and syrups [1,3,4].

These fruits are known as a rich source of dietary antioxidants mainly due to their high level of phenolic compounds, which primarily comprise ellagitannins, and flavonoids, including anthocyanins, and phenolic acids [5,6]. In recent years, these bioactive compounds have been associated with various health benefits, including antioxidant, prevention of inflammation disorders, cardiovascular diseases, or protective effects to lower the risk of various cancers. These properties and their presence in a majority of plant foods that are part of our daily diet, render phenolics very attractive molecules [1,2].

Phenolic compounds possess one or more aromatic rings with a conjugated aromatic system and one or more hydroxyl groups. They act as antioxidants, because they are able to donate an electron or a hydrogen atom to a free radical, what cause its conversion into an inoffensive molecule [1].

Important mechanism for anti-inflammatory action of polyphenols, especially flavonoids and anthocyanins is inhibition of eicosanoid generating enzymes—lipooxygenase (LOX) and cyclooxygenase (COX) [7]. Conversion products of polyunsaturated fatty acids, especially arachidonic acid (AA; 20:4 n-6) catalyzed by these enzymes may increase the risk of many diseases [8].

Epidemiological studies have shown a correlation between high fruit and vegetable consumption and the decreased incidence of cancers of various organs: lungs, larynx, throat, mouth, gastrointestinal tract, and pancreas [9]. Supplements made of a variety of vegetables and fruits can protect against the development of cancer as well. Numerous studies conducted in vitro or in animal model systems have confirmed that anthocyanins and anthocyanidins, mainly in the form of mixtures, have chemopreventive properties in relation to breast, skin, esophagus, lung, oral cavity, and gastrointestinal tract cancers [10,11,12,13].

There are studies considering beneficial properties of individual compounds from red raspberries related to their antioxidant action, but very important for dieticians and consumers is the entire pool of polyphenols. The total antiradical activity is not the sum of the action of the individual components, as various interactions, both synergistic and antagonistic, occur between the compounds [1,14].

Researchers mainly focus on analysis of antioxidant activity of crude or partially purified anthocyanins from raspberry fruit, or on comparison of composition of individual phenolic compounds between different raspberry varieties. In our study we evaluated the antioxidant and anti-inflammatory, activities, as well as cytotoxicity in vitro, of crude extracts made from raspberry pulp (RCE) and juice (JCE) as well as separated anthocyanin-rich fractions (RAFR and JARF) and other phenolic fractions (RPF and JPF).The purpose of our research was the anthocyanin and non-anthocyanin part extraction from raspberry fruit and juice and their relation in real conditions. As far as we know, for the first time the pro-health properties of crude extracts and separated anthocyanin fraction and non-anthocyanin fraction from these fruits have been compared. Similarly, there was no comparison of the type of LOX and COX-2 inhibition by the anthocyanin and polyphenol fractions separately.

## 2. Results and Discussion

### 2.1. Phenolic Content

Raspberries are very popular fruit in Poland and Europe, known in traditional medicine as a remedy for flu-like infections. Bioactive agents, especially phenolic compounds, are involved in these properties [15]. Due to their low stability, fresh raspberries are often processed into juice. In our research, the amount of polyphenols in the raspberry pulp was 270 mg/100 g FW, which was significantly higher than in the raspberry juice (192 mg/100 g FW). This indicates that, during the production of juice, approximately one third of the total content remains in the pomace, which is a waste material. Similarly, the total flavonoid and anthocyanin content in the fruit pulp was higher than in the juice, i.e., approximately 32% and 54%, respectively (Table 1).

These results corresponds well with values reported by other researchers. Pavlović et al. compared *inter alia* the abundance of phenolic compounds in different varieties of raspberries (*Rubus idaeus* L.) growing in Serbia. The content of phenolic compounds in the methanol extracts of the raspberries ranged from 243.47 mg/100 g for the variety Tulameen to 317.95 mg/100 g for the variety Yellow Meeker [16]. On the other hand, Stajčić et al. determined phenolic compounds at 304 mg/100 g for the variety Meeker and 265 mg/100 g for Willamette [17].

The content of phenolic compounds in different varieties of raspberry collected in the area of eastern Finland ranged from 192 to 359 mg/100 g. Among the raspberries grown in Lithuania, the greater amounts of phenolic compounds, 503.9 mg/100 g, were contained in the variety Glen Moy, including 130.6 mg/100 g of anthocyanins. The variety Polana contained 314.7 mg/100 g of phenolic compounds and 83.5 mg/100 g of anthocyanins [18]. The ratio of TFd in relation to TPh was 0.69 for RCE and 0.66 for JCE (Table 1). This implies that flavonoids are dominant phenolic components of the analyzed extracts. Similar results were obtained by Ĉetojević-Simin et al. for a Meeker raspberry pomace extract [19]. The anthocyanin content in the RCE sample was higher than in the JCE sample. Li et al. also used solid-phase extraction to extract an anthocyanin-rich fraction (ARF) from raspberries. They reported that the amount of ARF in 500 mg of a raspberry crude extract was 86.7 mg [20].

### 2.2. HPLC Identification of Phenolics and Anthocyanins

The aim of this study was not to perform accurate identification of the phenolic compounds present in raspberries and raspberry juice. For consumers, dieticians, and product quality, it is essential to assess the value of health benefits of the entire pool of phenolics rather than individual compounds. Qualitative and quantitative analysis of phenolic compounds was performed separately for the anthocyanin-rich fraction from raspberry pulp (RARF) and juice (JARF) purified on C-18 cartridges and the fraction of other phenolic compounds, also from raspberry pulp (RPF) and juice (JPF). Due to the existence of numerous derivatives of phenolic compounds, often in combination with various sugars, in this study, we determined the content of some compounds selected from the group of phenolic acids and flavonoids, based on the retention times and spectral analysis by UV-Vis.

In the present work, we identified four derivatives of cyanidin and one derivative of pelargonidin in the purified RARF from the raspberry fruit, while cyanidin-3-*O*-glucosylorutinoside was not detected in the JARF (Table 2). Probably, one of the sugar residues was disconnected during the purification or preparation of the samples. Our results correspond well with the results reported by other researchers. Literature data indicate that cyanidin and pelargonidin are the main anthocyanin aglycons presented in genus *Rubus* fruit. Pelargonidin derivatives are generally present in trace quantities, and derivatives of cyanidin are the main compounds, although the relationship is exactly opposite in the case of *Rubus pileatus* F. [14]. Guiné et al. detected delphinidin-3-*O*-glucoside in raspberry fruit, but these authors do not indicate the variety of the raspberries [21].

The chromatographic characteristics and content of the separated phenolic compounds are shown in Table 2. In our research, ellagic acid and its derivatives are the main phenolic acid in the phenolic fraction of the raspberry pulp (RPF) and juice (JPF). In the flavonoid group, there are derivatives of quercetin and kaempferol as well as (+) catechin and (−) epicatechin. Chromatograms are displayed in Supplementary Materials Figures S1 and S2.

Literature data shows the presence of glycoside derivatives of quercetin and kaempferol and a small amount of rutin and myricetin in raspberry fruit. From the group of flavan-3-ols, the presence of catechin, epicatechin, epigallocatechin, and procyanidin B1-B8 and C has been confirmed [22,23]. Among phenolic acids, ellagic acid and gallic acid, as well as smaller amounts of hydroxybenzoic, ferulic, protocatechuic, *p*-hydroxybenzoic acid, *p*-coumaric, salicylic, or caffeic acids have been mentioned as components of raspberry fruit [22,23,24]. From 27.5 mg/100 g DW of phenolic acids present in red raspberry, about 78% are derivatives of hydroxybenzoic acid—gallic acid and its dimer—ellagic acid [25]. Jakobek et al. identified ellagic acid in concentration 32 µg/g [20]. Soutinho et al. showed that the total hydroxybenzoic acids present in raspberry amounted to 14.48 µg/g GAE, with vanillic acid representing 75%. Hydroxycinnamic acids represented 37.62 µg/g GAE, with ferulic acid accounting for 79% [26]. 

The contents of phenolics and anthocyanins obtained with the spectrophotometric and HPLC methods are quite different. Essentially, the Folin-Ciocalteau method used to determine the total phenolic content may affect the result. The Folin-Ciocalteau reagent can react with sugars, ascorbic acid, proteins, amino acids, and copper and iron ions. These compounds in raspberry fruits can cause an increase in absorbance determinations. However, this method is still widely used for determination of the total phenolic compounds in the plant material and in food; therefore, it is easier to compare our results with those reported by other researchers. On the other hand, the use of the HPLC-method may lead to an underestimation of the quantities of phenolics and anthocyanins present in samples that contain various sugar derivatives of anthocyanidins, when used as a standard quantitative calculations. In addition, anthocyanin fractions purified on Sep-Pak^®^ C18 columns were analyzed by HPLC, and the purification process may lead to destruction thereof [27].

### 2.3. Antioxidant Potential

Different antioxidant compounds may act through different mechanisms, so using only one method is insufficient to fully evaluate the antioxidant capacity of foods [22,28]. The antioxidant activity of the analyzed samples was determined using three different methods. All analyzed fractions exhibited relatively high antioxidant activity determined with the DPPH method. The highest antiradical activity was determined for the extract obtained from the raspberry pulp (RCE). The value was almost a sum of the activity of the non-anthocyanin (RPF) and anthocyanin (RARF) fractions. Analogical proportions were found for the extracts prepared from the juice fractions (Table 3).

In both cases, the phenolic fraction activity reached approximately 60% of the activity of the crude extracts. Therefore, it can be concluded that it might be useful to use raw raspberry extracts as a protection against free radicals. In recent years, metal chelation has also been reported as a major contributor to cellular polyphenol antioxidant activity [14]. The ability to chelate Fe (II) ions of the crude extract obtained from the raspberry pulp was higher than the sum of these activities of the RPF and RARF. The same situation can be observed for the determined reducing power. Probably, this can be considered as a synergistic effect of all phenolic components of the extract. In the case of the raspberry juice, the reducing power of the crude extract was almost a sum of the activity of the separate fractions. Literature data confirm that polyphenolic antioxidants of red raspberries possess the ability to reduce ferric ions, acting as electron donors [14,15]. It is difficult to compare FRAP results of other researchers because of the use of a variety of different units, apart from the applied methods of extraction. The FRAP values, expressed as Trolox equivalents for Polana raspberry varied from 8.7 μmol TE/g FW to 12.88 μmol TE/g FW, depending on the year of harvesting [3]. These results are significantly higher than these obtained in this study, but this may be due to differences in the methodology used.

Antioxidant activity, measured by the DPPH assay, ranged from 64.14 ± 0.98 μmol TE/g FW (Rubin) to 127.59 ± 1.84 TE/g FW (Hollanda Boduru) in raspberry fruits [28]. In turn, lyophilized aqueous extracts of domesticated and wild ecotypes of raspberry fruits had a strong chelating effect on Fe^2+^, ranged from 0.934 to 1.008 Trolox equivalent. Also DPPH radical scavenging effect of the domesticated and 3 wild ecotypes of raspberry fruits were found as 0.899, 0.903, 0.617, and 0.628 trolox equivalents, and decreased in the following order: wild Yavuzlar ecotype > wild Yayla ecotype > Willamette ≈ wild Yedigöl ecotype. These extracts demonstrated powerful Fe^3+^ reducing ability ranged from 0.658 μg TE to 0.442 μg TE [24]. These results are in agreement with our results, taking into account that our results were expressed in mg/100 g fresh weight. The antioxidant potential (chelating power and reduction power) of RARF and JARF probably are the result of high concentration of cyaniding derivatives. In turn, higher ability to neutralize DPPH radicals of RPF and JPF may be due to presence of ellagic acid and (−) epicatechin, which have strong antioxidant properties [1].

### 2.4. Anti-Inflammatory Activity

**Figure 1:**
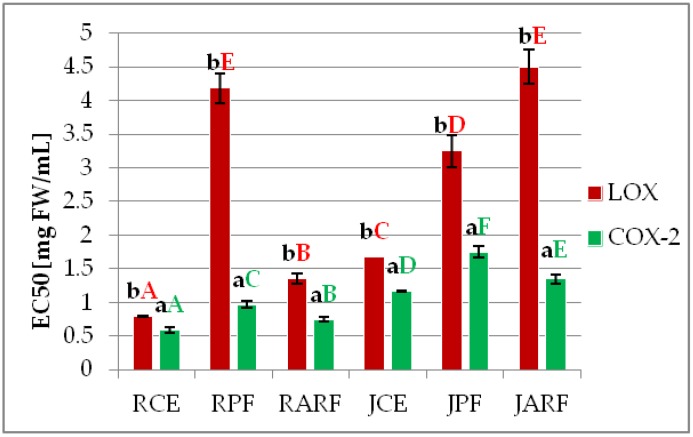
Anti-inflammatory activity of analyzed samples designated as LOX and COX-2 inhibitory activity—EC50 [mg FW/mL]. Abbreviations: RCE—raspberry crude extract, RPF—raspberry phenolic fraction, RARF—raspberry anthocyanin-rich fraction, JCE—raspberry juice crude extract, JPF—raspberry juice phenolic fraction, JARF—raspberry juice anthocyanin-rich fraction. Averages with different capital letters show significant differences between samples within one enzyme (red colour for LOX, green for COX-2) and with small letters show significant differences between enzymes (LOX and COX-2) inhibition by the same sample at *p* ≤ 0.05.

As shown in Figure 1, all analyzed samples possess anti-inflammatory potential, expressed as the ability to inhibit LOX and COX-2, which are enzymes involved in the inflammatory process. In the case of LOX inhibition, the lowest EC50 value was obtained for the RCE sample (0.79 mg FW/mL). For the RPE and JPE samples, the values were about 5.3- and 1.7-fold higher. Similar relations were observed for the extracts obtained from the juice and juice fractions. This is a clear evidence of synergism.

Knaup et al. showed that the extracted anthocyanins from blueberry and cranberry juice and chokeberry concentrate effectively inhibited the activity of soybean lipoxygenase-1 and human 5-lipoxygenase isolated from neutrophils [29]. Bräunlich et al. showed that both chokeberry extracts and purified fractions of anthocyanins and proanthocyanidins inhibited the activity of 15-LOX, and the effect was stronger for the fraction containing proanthocyanidins [30]. Similar results were obtained for anthocyanin extracts of three varieties of chokeberry (*A. melanocarpa*) and one of *A. prunifolia* which inhibited the activity of 15-LOX in the range of 8.5–17.4% at a concentration of 83.3 mg/mL [31]. The strong negative correlation for reduction power of analyzed extracts and their ability to inhibit LOX activity (EC50 value) was observed (−0.866)—Table S1.

For COX-2, the inhibitory effect was significantly stronger than for LOX. The highest enzyme inhibition was noted for crude extracts, but the differences from the separated fractions were not considerable. In all cases, the anthocyanin fractions exhibited higher inhibitory activity than the phenolic fractions (Figure 1). Several studies have shown that polyphenols, including anthocyanins from berries, may have anti-inflammatory properties [7,8,14]. A hexane extract of *Rubus jamaicensis* fruit showed a moderate ability to inhibit COX-2 (27.8–33.1%) at a concentration of 100μg/mL [14]. Anthocyanins from cherries and raspberries at a concentration of 125 μg/mL inhibited 45% of COX-1 activity and 47% of COX-2 activity. The inhibitory effect of anthocyanins contained in these fruits was comparable to that of ibuprofen and naproxen at a concentration of 10 µM [9]. Literature data shows that anthocyanin-rich fruits are effective in inhibiting COX-2 activity in vitro. Anthocyanins from *Amelanchier* spp., at a concentration of 100 ppm inhibited COX-2 activity in the range of 50–75%. For comparison, at the same concentration, aglycone cyanidin showed inhibition of 75%, and strongly inhibited the activity of COX-1 (96%) in contrast to the extract [32]. The strong negative correlation for chelating power of analyzed extracts and their ability to inhibit COX-2 activity (EC_50_ value) was observed (−0.829)—Table S1.

**Figure 2:**
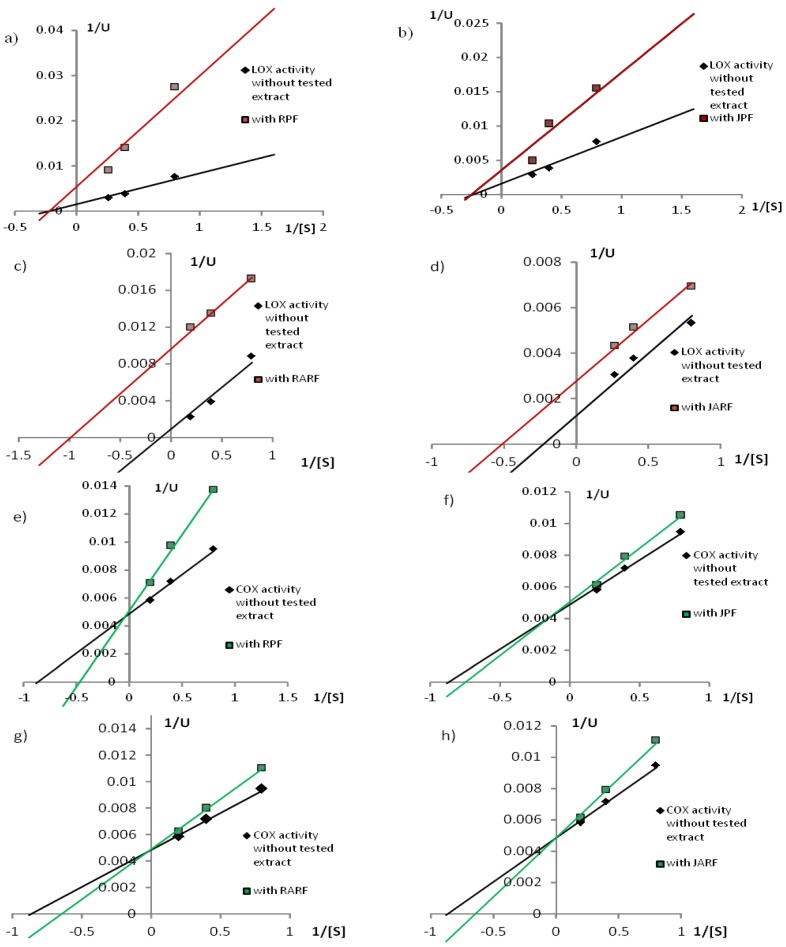
Mode of LOX (**a**–**d**) and COX (**e**–**h**) inhibition by analyzed extracts: RPF—raspberry phenolic fraction, RARF—raspberry anthocyanin-rich fraction, JPF—raspberry juice phenolic fraction, JARF—raspberry juice anthocyanin-rich fraction. Control—the activity of enzyme without tested extract.

The Lineweaver-Burk plot for lipoxygenase inhibition is shown in Figure 2a–d. In this work, RPF and JPF have been shown to inhibit LOX activity in a noncompetitive manner (Figure 2a,b) and anthocyanin fractions (RARF and JARF) inhibited it in an uncompetitive manner (Figure 2c,d). The uncompetitive type of anthocyanin inhibition over LOX is confirmed by the fact that the inhibition effect was the same regardless of whether the enzyme preincubation was applied with an inhibitor or not. In contrast, in the case of COX-2, both phenolic (RPF and JPF) (Figure 2e,f) and anthocyanin fractions (RARF and JARF) (Figure 2g,h) are competitive or mixed inhibitors. The Lineweaver-Burk plot for COX-2 inhibition is shown in Figure 2e–h.

The most common mechanism of inhibition of lipoxygenase activity by antioxidants is to inhibit the formation of lipid hydroperoxides by neutralizing the fatty acid radicals formed in the first LOX-catalyzed reaction step [33]. Another type of LOX inhibitors are redox-type inhibitors, which act by chelation of Fe (II) or reduction of the Fe(III) to Fe (II) [34] suggesting a competitive model. However, it has also been shown that LOX can be broken in a noncompetitive or mixed way (competitive/non-competitive). Some inhibitors do not show a linear relationship between the inhibitor concentration and the reaction rate, and a hyperbolic plot is obtained [8]. Knaup et al. analyzed the ability of various glycosides of delphinidine, cyanidine, peonidine, and malividine to inhibit LOX activity. All the analyzed compounds inhibited LOX in an uncompetitive manner, which confirms the results obtained in this work. Uncompetitive inhibitors bind to the enzyme-substrate complex without affinity for the free enzyme. As explained by these authors, when the inhibitor is attached to the enzyme-bound lipid radical species, the iron in the active center probably remains in a reduced form (inactivated) despite the radical disconnection in the next reaction step. This leads to a decrease in the pool of active enzyme molecules [29]. Such mechanism of action was also reported by other researchers. They showed that stilbene derivatives, such as resveratrol, structurally similar to curcumin, having a ketone moiety are effective COX-2 inhibitors [35].

### 2.5. Analysis of Cell Viability In Vitro—A Pilot Study

**Figure 3:**
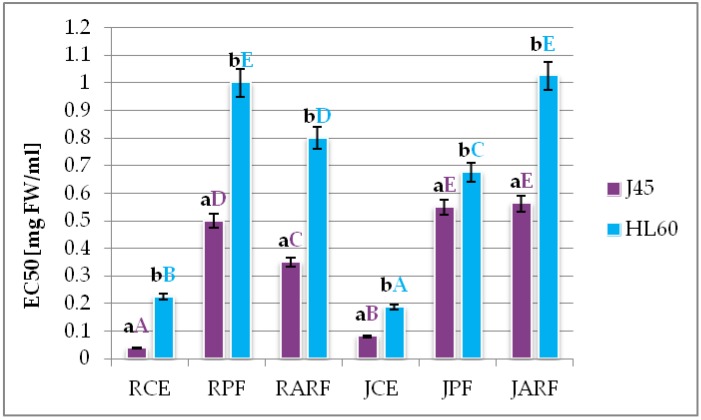
Cytotoxicity of analyzed extracts against J45 and HL60 cell lines [EC50 values]. RCE—raspberry crude extract, RPF—raspberry phenolic fraction, RARF—raspberry anthocyanin-rich fraction, JCE—raspberry juice crude extract, JPF—raspberry juice phenolic fraction, JARF—raspberry juice anthocyanin-rich fraction. Averages with different capital letters show significant differences between samples within one cell line (violet for J.45 and blue for HL60) and with small letters show significant differences between cell lines (J45 and HL60) viability inhibition by the same sample at *p* ≤ 0.05.

In this study, the analyzed extracts inhibited cell viability of both HL60 and J45 leukemia cells. The cytotoxic effect of the raspberry extract was dependent on the concentration and the cell line (Table S2). The J45 cell line was more sensitive, as the examined extracts inhibited the cell viability far more effectively. The value of the EC50 determined for the J45 line was significantly lower than that noted for the HL60 line (Figure 3).

The purified phenolic fraction from raspberries (RPF) inhibited the proliferation of J45 cells about twice as effectively as in the case of HL60. Exposure of the leukemic cells to increasing concentrations of the purified anthocyanin fraction from raspberry (RARF) caused a decrease in cell viability of both analyzed cell lines. On this basis, the concentration that inhibited cell viability by 50% (EC_50_) was determined. It was 0.35 mg FW/mL for line J45 and 0.8 mg FW/mL for line HL60.

The EC_50_ value of the HL60 line exposed to the raspberry juice extract (JCE) was estimated at 0.1875 mg FW/mL and was 2.3 times higher than for the J45 line. The non-anthocyanin fraction from the raspberry juice (JPF) significantly inhibited the viability of both cell lines. The determined EC50 values were 0.55 and 0.675 mg FW/mL for the J45 cell line and HL60, respectively. As a result of the exposure of the leukemic cells to the juice anthocyanin-rich fraction (JARF), 50% viability for line J45 was determined at a concentration of 0.5625 mg FW/mL, and the value for HL60 cells was almost twice as high. Additionally, stronger negative correlation was found for antioxidant activity of analyzed extracts and their ability to inhibit viability of the J45 cell line than HL60 cell line. The strongest correlation for reducing power and EC_50_ value was observed for J45 cell line (r^2^ = −0.905)—Table S1.

The investigated cell lines represent different types of leukemia (J.45—acute non-tumorigen T-lymphoblastoid cell leukemia, HL60 tumorigenic acute promyelocytic leukemia, which contains mainly promyelocytes). Additionally, in J.45 cells the p53 protein, which is responsible for cell cycle control is defective, whereas in HL60 cells is absent. These differences may lead to different responsiveness of J.45 and HL60 in the cytotoxicity tests [36]. A similar tendency (higher toxicity in relation to the J45 than HL60 lines) was observed in earlier studies [37]. Literature data confirm our results, indicatind that J.45 cells are more sensitive to chemopreventive agents, such as anthocyanins, than HL60 cells. The IC_50_ for Jurkat (J.45) cells treated with cyanidin-3-*O*-β- glucopyranoside was seen at a concentration of 174.9 µg/mL, whereas only a slight decrease in the number of viable cells was observed for HL-60 (IC_50_ value did not fall within the range of concentrations tested) [36].

Recent publications also show the anti-tumor potential of berries in vitro. Out of the 10 ethanolic extracts from berries, the blueberry extract was the most effective in inhibition of the growth of human leukemia (HL 60) and colon carcinoma (HCT116) cells in vitro [38]. Other authors demonstrated that cyanidin-3*-O*-rutinoside isolated and purified from the black raspberry fruit induced apoptosis upon generation of peroxides in tumor HL60 cells, to some extent dependent on the dose and duration of the action. Most importantly, these authors did not observe a prooxidative effect in relation to normal human peripheral blood mononucleated cells [39]. Dai et al. analyzed the effect of extracts of blackberry fruit (puree and lyophilizate) and cyanidin-3-*O*-glucoside, which is the major anthocyanin in these fruits (71%), on the viability of tumor cell lines HT-29 (colon carcinoma), MCF-7 (breast cancer), and HL60 cells (leukemia). The extract obtained from the blackberry puree exhibited over two-fold greater cytotoxicity to line HL60 than the freeze-dried extract. In contrast, cyanidin-3-*O*-glucoside at a concentration present in the fruit virtually showed no cytotoxicity [40]. This confirms the results obtained in this work. Purified anthocyanin fractions (RARF and JARF) inhibited cell viability less potently than the entire pool of the extracted compounds (crude extracts). A similar result was observed for the purified phenolic fractions (RPF and JPF). Sharif et al. showed that chokeberry juice inhibited proliferation of tumor cells of acute lymphoblastic leukemia (Jurkat lines) and contributed to cell cycle arrest in the G2 phase, resulting in a statistically significant increase in the number of apoptotic cells even at a concentration of 0.2% (*v*/*v*) [41].

## 3. Materials and Methods

### 3.1. Material

Raspberries (1 kg) were blended for 1 min in a blender (Braun GmbH, Kronberg im Taunus, Germany), heated in a shaking incubator (INCU-Shaker MINI, Benchmark Scientific, Sayreville, NJ, USA) to a temperature of 45 °C. Next raspberry mash was cooled and divided into two parts. One part—raspberry puree was stored at −20 °C and used for extract preparation. The second part was squeezed (Eujuicers.com 8004 squeezer, Omega, Prague, Czech Republic) and centrifuged at 7000 rpm for 15 min at 4 °C. The supernatant (juice) was stored at −20 °C and used for analyses.

### 3.2. Isolation of Phenolic Compounds

The extraction of phenolic compounds was performed according to the method described by Rodriguez-Saona and Wrolstad [42]. Fifteen g of raspberry pulp or juice were extracted for 1 h three times with 30 mL of 90% acidified methanol (0.1% *v*/*v* HCl). After each cycle, the extracts were centrifuged for 15 min at 9000× *g* at 4 °C and filtered. Combined supernatants were made up with acidified methanol to a volume of 100 mL. Ten mL of the crude extract was used for further analysis and next 90 mL of RCE and JCE were evaporated using a vacuum evaporator (40 °C, 700 MPa) and dissolved in 5 mL of deionized acidified water (0.1% *v*/*v* aqueous solution of HCl) and then fractionated using a procedure described by Rodriguez-Saona and Wrolstand [42]. The extracts were passed through Supelco C-18 cartridges (Sigma-Aldrich, Poznań, Poland) activated with acidified methanol followed by 0.1% HCl (*v*/*v*) in deionized water. Anthocyanins were adsorbed onto the column while carbohydrates, acids, and other water-soluble compounds were removed by flushing with 0.1% HCl. Next the cartridges were washed with ethyl acetate to remove phenolic compounds other than anthocyanins. The ethyl acetate fractions were evaporated using a rotary evaporator at 40 °C and the solids were dissolved in methanol and used for further analysis as a purified phenolic fraction (RPF and JPF). Anthocyanins were then recovered with methanol containing 0.1% HCl (*v*/*v*). The methanol fractions were evaporated using a rotary evaporator at 40 °C and the solids were used for further analysis as a purified anthocyanin-rich fraction (RARF and JARF).

### 3.3. Total Phenolic Content

The analysis was carried out with the Folin-Ciocalteau method [43]. To 0.1 mL of properly diluted extract 0.1 mL of distilled water and 0.4 mL of Folin-Ciocalteau reagent (diluted with distilled water 1:5) were added. After three minutes, 2 mL of a sodium carbonate solution (10%) were added and vigorously mixed. The absorbance was measured after 30 min, at 725 nm using a Lambda 40 UV-Vis spectrophotometer (PerkinElmer Inc. Waltham, Massachusetts, USA.). The total phenolic content was expressed in mg/100 g fresh weight (FW) as gallic acid equivalent through the calibration curve of gallic acid.

#### HPLC Analysis of Phenolic Compounds

The samples were analyzed with a Varian ProStar HPLC System separation module (Varian, Palo Alto, CA, USA) equipped with a Varian ChromSpher C18 reverse phase column (25 mm 4.6 mm) and a ProStar DAD detector. The column thermostat was set at 40 °C. The mobile phase consisted of 4.5% acetic acid (solvent A) and 50% acetonitrile (solvent B), and the flow rate was 1 mL min^−1^. The gradient elution was used as follows: 0 min 100% A, 5 min 100% A, 20 min 90%, 35 min 80% A, 50 min 70% A, 60 min 55% A, 70 min 0% A, and 85 min 0% A. At the end of the gradient, the column was washed with 100% acetonitrile and after that equilibrated to the initial condition for 10 min. Spectrum analysis and a comparison of their retention times with those of the standard compounds identified the phenolics in the sample. Quantitative determinations were carried out with the external standard calculation using calibration curves of the standards at concentrations ranging from 0.05–5 mg/mL (r^2^ = 0.999). Detection was performed at 280 nm for gallic acid, (+)-catechin and (−)-epcatechin, 320 nm for quercetin and kaempferol and 260 nm for ellagic acid [44]. Phenolics were expressed in µg per g of fresh weight (FW).

### 3.4. Flavonoid Content

The total flavonoid content was measured with an aluminum chloride colorimetric assay. To 0.5 mL of properly diluted acidified methanolic extract 0.5 mL of 2% solution of AlCl_3_ × 6H_2_O in methanol was added. After 10 min the absorbance was measured against methanol at 430 nm. The flavonoid concentration was expressed in mg/100 g fresh weight (mg/100 g FW) as quercetin equivalent [45].

### 3.5. Anthocyanin Content

The total anthocyanin content in the obtained extracts was determined using the pH differential method [46]. Two dilutions of the same sample were prepared in a 0.025 M potassium chloride solution and in a 0.4 M sodium acetate solution, adjusted respectively to pH 1.0 and 4.5 with HCl. The absorbance of each dilution was measured at 520 and 700 nm using a distilled water as a blank. Absorbance (A) was calculated as follows:

A = (A_520_ − A_700_)_pH1.0_ − (A_520_ − A_700_)_pH4.5_

The anthocyanin concentration (mg/L) was calculated using the following formula:

Anthocyanin content = (A × MW × DF × 1000)/(ε × 1)

where MW is the molecular weight of cyanidin-3-glucoside (449.2 gmol^−1^), DF is the dilution factor, and ε is the molar extinction coefficient of cyanidin-3-glucoside (ε = 26 900 L cm^−1^ mol^−1^). Total anthocyanin was calculated in the sample as mg per 100 g of fresh weight (FW).

#### HPLC Analysis of Anthocyanins

RARF and JARF were used for the quantitative analysis of anthocyanins via HPLC. The samples were analyzed with a Varian Pro-Star HPLC System separation module equipped with a Nucleosil 100-5 C18 reverse-phase column (250 × 4.6 mm, particle size 5 µm, pore size 100 A), and a ProStar DAD detector. The column thermostat was set at 25 °C. An elution gradient with 4.5% formic acid in water as solvent A and 2% formic acid (*v*/*v*) in acetonitrile as solvent B were used with the following profile: 0–30 min: 100–73% A, 30–35 min: 73–87% A, and 35–55 min: 87–0% A with a flow rate 1 mL min^−1^. At the end of the gradient, the column was washed with acetonitrile and equilibrated to the initial condition for 10 min. The injection volume of the sample was 10 μL and detection was carried out at the wavelength 520 nm. Quantitative determinations were carried out with an external standard calculation, using calibration curves of the standards (cyanidin-3.5-di-*O*-glucoside, cyanidin-3-*O*-glucoside, cyanidin-3-*O*-rutinoside, cyanidin, pelargonidin-3-*O*-glucoside, delphinidin, malvidin-3.5-di-*O*-glucoside), each of the following concentrations: 1.25, 2.5, 5, 10, and 20 µgmL^−1^ dissolved in 0.1% HCl in 100% methanol (r^2^ = 0.9998). Anthocyanins were separated and analyzed by comparing retention times and UV–Vis absorption spectra [47].

### 3.6. Antioxidant Activities

#### 3.6.1. Free Radical Scavenging Assay

Free radical scavenging activity was measured using 1,1-diphenyl-2-picrylhydrazyl (DPPH) [48] as a source of free radicals. 0.08 mL of properly diluted extracts was mixed with a 1.92 mL 6 × 10^−5^ M solution of DPPH^•^ in methanol and then decrease of the absorbance was measured at λ = 515 nm over 2.5 min against a blank sample containing methanol. The ability of the analyzed extracts to quench DPPH free radicals was calculated as follows:

scavenging % = [(A_C_ − A_A_)/A_C_] × 100

where: A_C_—absorbance of the control (the solvent instead of the extract) at 0 min, A_A_—absorbance of the sample after 2.5 min. The antioxidant activity was expressed as µmol of Trolox per 100 gram of fresh weight (FW) (TE—Trolox equivalent). The standard curve was prepared in the concentration range of 0–1500 μmol of Trolox (r^2^ = 0.978).

#### 3.6.2. Chelating Power

Iron chelating activity was determined with the colorimetric method of Guo et al. [49]. To the extract samples (0.1 mL of the extract diluted to 1 mL with methanol) 0.02 mL of a 2 mM FeCl_2_ solution and 0.04 mL 5 mM ferrozine were added. The mixture was shaken vigorously and left standing at room temperature for 10 min. Absorbance of the solution was then measured spectrophotometrically at 562 nm against a blank sample (0.1 mL of the extract and 1.06 mL of methanol to take into account the impact of the color of the extract). The control sample contained 1 mL of methanol, 0.02 mL of a 2 mM FeCl_2_ solution, and 0.04 mL of 5 mM ferrozine; methanol was a blank sample for this measurement. The percentage of inhibition of ferrozine—Fe^2+^ complex formation was calculated using the following formula:

% inhibition = (1 − A_A_/A_C_) × 100

where: A_C_—absorbance of the control (the solvent instead of the extract), A_A_—absorbance of the sample. Chelating power was expressed as EDTA equivalent in µg EDTA per 100 g of fresh weight (FW). The standard curve was prepared in the concentration range 0–15 μgmL^−1^ of EDTA (r^2^ = 0.996).

#### 3.6.3. Ferric Reducing Power (FRAP)

Reducing power was determined using the method described by Pulido et al. [50]. Properly diluted extracts (0.5 mL) were mixed with phosphate buffer (0.5 mL, 200 mmol L^−1^, pH 6.6) and 0.5 mL of a 1% aqueous solution of potassium ferricyanide K_3_[Fe(CN_6_)].The mixture was incubated at 50 °C for 20 min. Then 0.5 mL of 10% (*w*/*v*) trichloroacetic acid was added and obtained mixture was centrifuged at 25× *g* for 10 min. To the 1 mL of upper layer of the solution 1 mL of distilled water and 0.2 mL of 0.1% (*w*/*v*) FeCl_3_ wa added and then absorbance was measured at 700 nm. Reducing power was expressed as µmol of Trolox per 100 gram of fresh weight (FW).

### 3.7. Anti-Inflammatory In Vitro Activity

#### 3.7.1. LOX Inhibitory Activity

The activity of lipoxygenase (LOX) inhibitors was measured spectrophotometrically according to the method described by Axelroad et al. [51]. Lipoxygenase activity was determined at a temperature of 25 °C by measuring the increase in absorbance at 234 nm over a 3 min period. The reaction mixture contained 1/15 M phosphate buffer pH 7.0, 0.02 mL of a LOX solution (167 U/mL), and 0.025 mL of different concentrations of inhibitor (crude extract or purified phenolic or anthocyanin fraction). The mixture was incubated for 3 min and the reaction was initiated by adding 0.04 mL of 2.5 mmol/L linoleic acid. One unit of LOX activity was defined as an increase in absorbance of 0.001 per minute at 234 nm. The corresponding control contained the same concentration of the enzyme with the absence of the inhibitor. An extract concentration (mg FW/mL) providing 50% inhibition (EC50) was obtained by plotting the inhibition percentage against sample concentrations.

#### 3.7.2. COX-2 Inhibitory Activity

Cyclooxygenase-2 activity was determined spectrophotometrically at 610 nm by measuring the activity of the COX peroxidase subunit using NNN′N′-tetramethyl-*p*-phenylenediamine (TMPDA) as an electron donor. In the presence of arachidonic acid, oxidation of TMPDA and formation of a blue product takes place. 100 mM Tris-maleate buffer pH 6.5 was prepared (own modification in order to reduce autoxidation TMPDA—Tris-HCl buffer, pH 8.0 was used in the original). 40 µL of the COX-2 enzyme was incubated at room temperature for 1 min with the Tris-maleate buffer containing 1 µM of heme; next, 40 μL of 17 mM TMPDA in DMSO (own modification—in the original water solution was used) were added. The reaction was initiated by addition of 40 µL of 10 mM arachidonic acid and the change in absorbance was measured over 0.5 min relative to the blank sample containing the buffer alone. One unit of COX activity was defined as an increase in absorbance of 0.001 per min. Changes in absorbance were measured after 3 min of incubation of the enzyme with 0.01 mL of different concentrations of CE or ARF or PF. The amount of Tris-maleate buffer pH 6.5 to the total reaction volume was 1 mL. The blank sample contained Tris-maleate, pH 6.5, and a suitable amount of the extract (an inhibitor) in order to eliminate the influence of the color [52]. An extract concentration (mg FW/mL) providing 50% inhibition (EC50) was obtained by plotting the inhibition percentage against sample concentrations.

#### 3.7.3. Determination of the Type of LOX and COX-2 Inhibition

The double-reciprocal Lineweaver-Burk plot of 1/V versus 1/S was plotted to determine inhibition type of LOX and COX-2 by anthocyanin-rich fraction and phenolic fraction from raspberry pulp (RPF, RARF) and juice (JPF and JARF).

### 3.8. Cell Lines

Two cell lines from the European Collection of Cell Cultures (ECACC) were used:

- HL-60—Human Caucasian promyelocytic leukemia (ECACC No. 98070106).
- J45.01—Human acute T cell leukemia (ECACC No. 93031145). The cells at the density 0.5 × 10^6^ cells/mL were incubated in an air atmosphere humidified with 5% CO_2_ for 24 h at 37 °C in an incubator (Cellstar, Cleveland, OH, USA). The growth medium consisted of RPMI 1640 medium (Sigma, St. Gallen, Switzerland), heat-inactivated fetal calf serum (20% for HL-60 and 10% for J.45), 2 mM L-glutamine and antibiotics: penicillin at a concentration of 100 U/mL, streptomycin at a concentration of 100 μM/mL, and amphotericin B at a concentration of 2.5 μg/mL (Sigma).

#### 3.8.1. Preparation of Extracts for Cytotoxicity Assay

Raspberry pulp (10 g) and juice (10 g) were extracted three times with 15 mL of acidified 90% ethanol (0.1% *v*/*v* ethanolic HCl solution). After each extraction, samples were centrifuged for 15 min in 9000× *g* at 4 ° C and filtered. The supernatants were combined and made up with acidified ethanol to a volume of 50 mL. The crude extract was evaporated using a vacuum evaporator (40 °C, 700 MPa) and dissolved in 5 mL of acidified deionized water (0.1% *v*/*v* aqueous HCl solution). The aqueous extract was then fractionated using Sep-Pak^®^ C18 columns according to the scheme described in Section 3.2. After evaporation, the anthocyanin fraction was dissolved in 2 mL of water and the phenolic fraction in 2 mL of ethanol.

#### 3.8.2. In Vitro Cytotoxicity Assay—A Pilot Study

The trypan blue staining method was employed for preliminary evaluation of the effects of the extracts on the viability of the leukemia cells. After 24-hour incubation at 37 °C, the cell lines were exposed to different amounts of water or ethanolic extracts, respectively and incubated further for 24 h. The final concentration of ethanol was thereby reduced to 1% in the assays: this concentration of ethanol did not affect cell viability. Next, the cells were washed with PBS and centrifuged at 800 rpm for 10 min, and then PBS was removed by aspiration. Then, 10 μL of cell suspensions were incubated for 5 min with 10 μL 0.4% of the trypan blue solution (BioRad Laboratories, Inc., Hercules, CA, USA) and counted on a TC10™ Automated Cell Counter (Bio-Rad Laboratories, Inc.,Hercules, CA, USA). The cytotoxicity was determined as percentage of living cells compared to the control. If a certain concentration of analyzed extract reduced cell viability compared to the control, then that concentration was considered to be cytotoxic. These activities were evaluated by the EC_50_ (effective concentration of 50% cell viability) values, which were expressed as mg of analyzed extracts/mL. Each experiment was repeated three times.

### 3.9. Statistical Analysis

All experimental results were means and were performed in triplicate (three extracts and three measurements for each extract) and the data in the tables and figures represent mean values ± standard deviation (*n* = 9). The results were evaluated for statistical significance using univariate analysis of variance (ANOVA) with Statistica 13.0 software (StatSoft, Inc., Tulsa, OK, USA) and Tukey’s post hoc test. Differences were considered significant at *p* = 0.05.

## 4. Conclusions

Berry fruits are known to be rich sources of bioactive compounds such as phenolic acids, flavonoids and anthocyanins which have proven positive effect on human health. Diet rich in those natural supplements used as auxiliary medication is often an object of research and new products of natural origin are developed. Thus, based on obtained results, it can be concluded that crude raspberry extracts possess higher anti-oxidant, anti-inflammatory, and cytotoxic activities in comparison to purified anthocyanin or non-anthocyanin fraction, both for raspberry pulp and juice. The data (Lineweaver-Burk plots of LOX and COX-2 inhibition) indicates that the bioactive compounds of the different fractions of raspberry act via different mechanisms. Phenolic fractions (RPF and JPF) acts as noncompetitive inhibitors of lipoxygenase and competitive inhibitors of cyclooxygenase-2. In turn, anthocyanin fractions (RARF and JARF) inhibit LOX in uncompetitive manner. More research is needed to accurately determine the mechanisms of action and to analyze the type of interactions between individual raspberry antioxidants (synergism, antagonism or no interactions). The conducted study of the effect of analyzed extracts on leukemia cells viability is only preliminary. It also remains unknown whether the cell mortality is related to apoptosis or necrosis. Identification of cell compartment which is a target for plant-origin antioxidants may be very helpful in understanding not only deleterious but also beneficial effects of phenolic acids, flavonoids and anthocyanins. Further analysis are needed to check exact mechanism of cytotoxicity, which requires specific biochemical tests.

## Figures and Tables

**Table 1:** Total phenolic, flavonoid and anthocyanin content of raspberry and raspberry juice crude extracts (mg/100 g FW).

Sample	TPh (GAE mg/100 g)	TFd (QE mg/100 g)	TAc (CyGE mg/100 g)	TFd/TPh
RCE	270 ± 17.9 ^b^	186.8 ± 1.18 ^b^	58.7 ± 3.5 ^b^	0.69
JCE	192.04 ± 5.7 ^a^	127.07 ± 1.03 ^a^	42.9 ± 0.6 ^a^	0.66

^a,b^: means ± SD in the same column differ significantly at level *p* ≤ 0.05. Abreviations: RCE—raspberry crude extract, JCE—juice crude extract, TPh—total phenolic, TFd—total flavonoid and TAc—total anthocyanin contents; GAE –gallic acid, QE—Quercetin and CyGE—cyanidin-3-glucoside equivalents. Values represent means SD of three measurements (*n* = 3).

**Table 2:** Identified compounds concentration in anthocyanin rich fraction (ARF) and other phenolic fraction (PF) from raspberry pulp (R) and juice (J) determined by HPLC method.

**Identified Compound**	Concentration [µg/g FW] *	
	**RARF**	**JARF**
Cyanidin-3-*O*-sophoroside	332.0 ± 16	296 ± 25.6
Cyanidin-3-*O*-glucosylrutinoside	56.0 ± 2.7	nd.
Cyanidin-3-*O*-glucoside	42.12 ± 2	66.6 ± 5.76
Cyanidin-3-*O*-rutinoside	32.76 ± 1.6	5.55 ± 0.48
Pelargonidin-3-*O*-glucoside	4.68 ± 0.23	2.96 ± 0.25
**Identified compound**	Concentration [µg/g FW] *	
	**RPF**	**JPF**
Ellagic acid	66.96 ± 0.84	42.68 ± 0.53
Ellagic acid derivative	75.12 ± 0.94	47.68 ± 0.59
Gallic acid	9.63 ± 0.48	8.08 ± 0.404
Quercetin -based flavonol	25.13 ± 0.71	13.7 ± 0.68
Kaempferol -based flavonol	44.45 ± 2.22	19. 22 ± 0.96
(+) Catechin	8.08 ± 0.404	9.63 ± 0.48
(−) Epicatechin	61.18 ± 3.6	11.21 ± 0.36

Abbreviations: RARF—raspberry anthocyanin-rich fraction, JARF—juice anthocyanin-rich fraction, RPF—raspberry phenolic fraction, JPF—juice phenolic fraction, nd.—not detected. Values represent means SD of three measurements (*n* = 3). *—µg per g of fresh weight (FW).

**Table 3:** Antioxidant capacity of analyzed samples, measured by different complementary assays.

Sample	DPPH µM TE/100 g FW	Chelating Power mg EDTA/100 g FW	Reducing Power µM TE/100 g FW
RCE	588.9 ± 5.5 ^f^	16.9 ± 0.23 ^e^	1912.0 ± 1.78 ^f^
RPF	363.0 ± 3.4 ^e^	10.9 ± 0.56 ^b,c^	623.0 ± 8.46 ^b^
RARF	207.7 ± 4.4 ^d^	11.3 ± 0.28 ^c^	987.7 ± 5.52 ^d^
JCE	183.0 ± 6.7 ^c^	13.8 ± 0.22 ^d^	1146.1 ± 0.82 ^e^
JPF	166.3 ± 3.76 ^b^	5.9 ± 0.75 ^a^	664.8 ± 9.20 ^c^
JARF	107.8 ± 1.5 ^a^	9.9 ± 0.10 ^b^	510.9 ± 5.80 ^a^

^a–f^: means ± SD with different letters in the same column are statistically significant at *p* ≤ 0.05. Abbreviations: RCE—raspberry crude extract, RPF—raspberry phenolic fraction, RARF—raspberry anthocyanin-rich fraction, JCE—juice crude extract, JPF—juice phenolic fraction, JARF—juice anthocyanin-rich fraction.

## Supplementary Materials

The following are available online, Figure S1: HPLC chromatograms of anthocyanin fractions of (A) raspberry anthocyanin-rich fraction (RARF) and (B) juice anthocyanin-rich fraction (JARF), Figure S2: HPLC chromatograms of phenolic fractions of A) raspberry phenolic fraction (RPF) and B) juice phenolic fraction (JPF). Table S1: Correlation between antioxidant activity and inhibitory and antiproliferative activity of tested extracts, Table S2: Cell viability [%] of J.45 and HL60 cell lines exposed to increasing amounts [microliters] of analyzed extracts.
