# Supplementary material for: Antioxidant, Anti-Inflammatory, and Postulated Cytotoxic Activity of Phenolic and Anthocyanin-Rich Fractions from Polana Raspberry (Rubus idaeus L.) Fruit and Juice—In Vitro Study
Source: Molecules. 2018 Jul 21;23(7):1812. doi: 10.3390/molecules23071812 (PMC6099503; doi:10.3390/molecules23071812)
Supplement: Supplementary file 1 [file molecules-23-01812-s001.docx]

**Supplementary material: Antioxidant, anti-inflammatory, and postulated cytotoxic activity of phenolic and anthocyanin-rich fractions from Polana raspberry (*Rubus idaeus* L.) fruit and juice – *in vitro* study**

**Urszula Szymanowska, Barbara Baraniak and Anna Bogucka-Kocka**

| 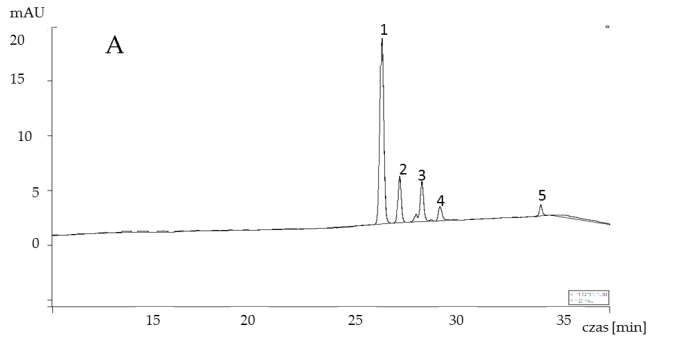 | 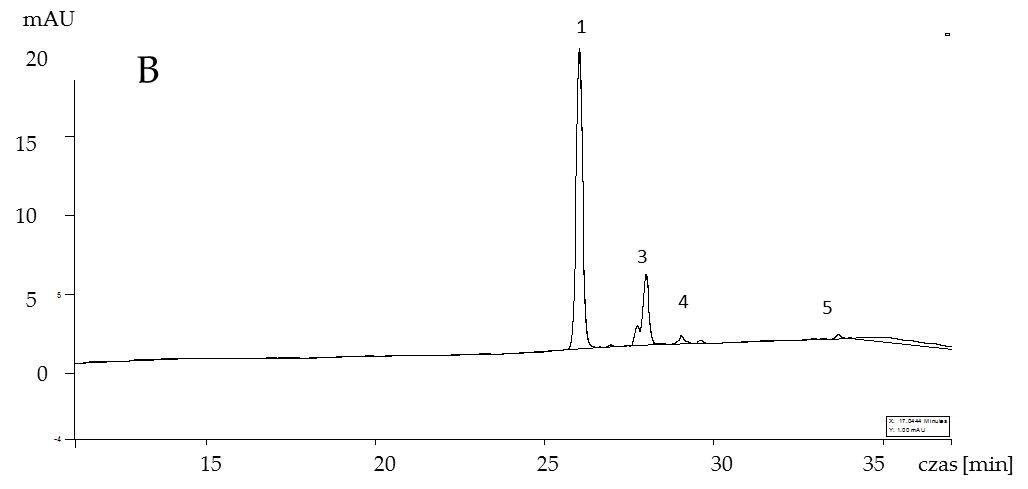 |
| --- | --- |

Fig S1. HPLC chromatograms of anthocyanin fractions of A) raspberry anthocyanin-rich fraction (RARF) and
B) juice anthocyanin-rich fraction (JARF). 1 - cyanidin-3-*O-*sophoroside, 2 - cyanidin-3*-O-*glucosylrutinoside, 3 - cyanidin-3*-O-*glucoside, 4 - cyanidin-3*-O-*rutinoside, 5 - pelargonidin-3*-O-*glucoside


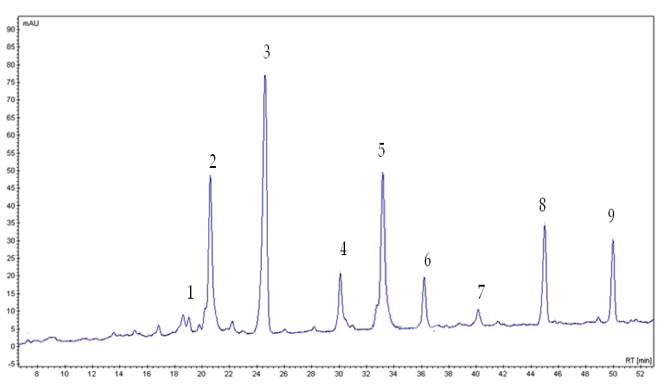

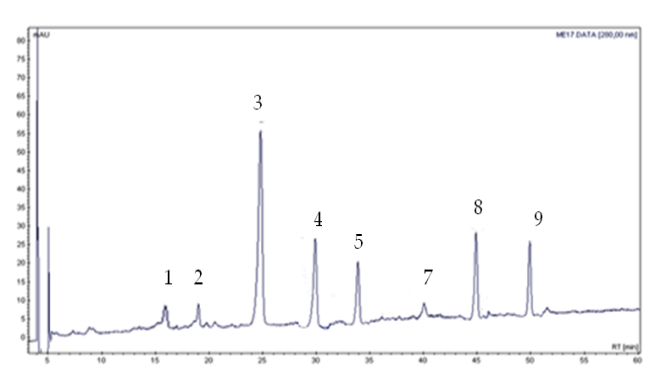


Fig.S2. HPLC chromatograms of phenolic fractions of A) raspberry phenolic fraction (RPF) and B) juice phenolic fraction (JPF). 1 - (+) catechin, 2 - (-) epicatechin, 3 – ellagic acid, 4, 5 -- ellagic acid derivatives, 7- gallic acid, 8 -kaempferol -based flavonol, 9 - quercetin -based flavonol

Table S1. Correlation between antioxidant activity and inhibitory and antiproliferative activity of tested extracts

| Antioxidant activity | LOX [EC50] | COX [EC50] | HL60 [EC50] | J45 [EC50] |
| --- | --- | --- | --- | --- |
| DPPH | -0.446 | -0.711 | -0.397 | -0.550 |
| CHELATING POWER | -0.665 | -0.829 | -0.634 | -0.883 |
| REDUCTION POWER | -0.866 | -0.679 | -0.816 | -0.905 |

Table S2. Cell viability [%] of J.45 and HL60 cell lines exposed to increasing amounts [microliters] of analyzed extracts

| SAMPLE [µL] | Cell viability [%] | | | | | | | | | | | |
| --- | --- | --- | --- | --- | --- | --- | --- | --- | --- | --- | --- | --- |
|  | RCE | | RARF | | RPF | | JCE | | JARF | | JPF | |
|  | J.45 | HL60 | J.45 | HL60 | J.45 | HL60 | J.45 | HL60 | J.45 | HL60 | J.45 | HL60 |
| C | 100 | 100 | 100 | 100 | 100 | 100 | 100 | 100 | 100 | 100 | 100 | 100 |
| 1 | 55.95 | 90.25 | 90 | 97 | 97 | 98 | 90 | 98.8 | 93.34 | 98.34 | 97.5 | 98.25 |
| 5 | 34.26 | 87.15 | 63 | 95.67 | 93 | 97 | 63 | 90.5 | 95 | 87.75 | 97 | 95 |
| 10 | 24.85 | 42 | 34 | 80 | 55 | 96 | 29.5 | 70.5 | 90.34 | 89.75 | 91 | 92 |
| 15 | 15.25 | 22 | 21 | 52.85 | 9.85 | 75 | 21 | 52 | 80.67 | 74.67 | 63.25 | 90.5 |
| 20 | 12 | 15 | 19 | 45.12 | 3 | 51 | 16 | 40 | 62.34 | 71.34 | 52 | 69.85 |
| 30 | 10 | 6 | 16 | 30 | 2 | 10 | 5 | 29.8 | 53.1 | 68.34 | 32 | 42 |
| 50 | 2 | 3.8 | 2 | 12 | 1 | 5 | 1 | 5 | 25 | 39.83 | 10 | 18 |
